# Progress in Bacteriology

**Published:** 1904-02-20

**Authors:** 


### PROGRESS IN BACTERIOLOGY.

**Acute Rheumatic Fever.**—W. V. Shaw[1] has conducted a research in order to substantiate the statements of former observers that the diplococcus rheumaticus is capable of exciting a specific fever with the gross anatomical lesions of rheumatic fever. Briefly, our knowledge of the bacteriology of rheumatic fever is as follows:—Klebs, Popow, Netter, and Dana have described a streptococcus. Achalme, Thiroloix, Bettencourt, and Hewlett found an anærobic bacillus. Hiva, Triboulet, Cyon, Apert, and Popoff attribute the disease to a diplococcus which is almost certainly identical with the diplococcus described by Wassermann, Westphal, and Malkoff, who noted its growth in alkaline media and its tendency to assume a streptococcal form. The existence of such a diplococcus in cases of rheumatic fever has been confirmed by Poynton and Paine, and by Beaton and Ainley Walker. The micrococcus grows on alkaline beef broth, 1 per cent. alkaline peptone agar, glycerine agar, and blood agar. On the last-named it appears in small discrete, colourless, transparent colonies, which gradually coalesce to form a film converting in the second day the red blood into a rusty brown or greenish-brown background. Gelatin is not liquified, neutral milk is coagulated in 48 hours. In broth no turbidity is caused but a flocculent precipitate falls. The cocci measure ·5 to 1$\mu$ in diameter and are found in pairs or short chains. In cultures 48 hours old involution forms begin to appear, diplo-baccilli 2·5 to 3$\mu$ long by ·5 to 1$\mu$ broad, forming short chains. These resemble the involution forms of the streptococcus scarlatinæ described by Gordon. The micrococcus may be recovered from the blood, urine, tonsils, or joint fluids of cases of rheumatic fever. They are also present in cases of chorea, endocarditis, and in rheumatic nodules. Shaw tested strains of the diplococcus supplied by Wassermann, by Ainley Walker and by Poynton. The differences between these were very small and on rabbits and monkeys they produced when injected intravenously myocarditis, pericarditis, endocarditis, arthritis, pleurisy, iritis. The anatomical nature of these lesions was identical with those found in rheumatic fever. It has been claimed that rheumatic fever is an attenuated pyæmia and that it may be excited by many of the pyogenetic cocci, and it has also been said that vegetative growths on the endocardium are to be found in healthy rabbits. Shaw however believes that the diplococcus of rheumatism is a specific organism, and this would seem to be supported by the fact that it never gives rise to suppuration, and its lesions are of a peculiarly distinctive character. Shaw[2] says that in none of a large number of rabbits artificially infected with such organisms as B. coli B. typhosus, B. tuberculosis, streptococcus, pneumococcus, etc., and subsequently killed were endocardial changes found so that the power of exciting such changes must be a distinctive quality of the rheumatic diplococcus. These results differ slightly from those recorded by Poynton,[3] who stated that the maximum growth was obtained anærobically in a medium of milk and bouillon acidified with lactic acid. Poynton laid stress on the fact that the diplococcus perished quickly in the tissues, although leaving behind it lasting changes. He described, too, a solitary coccal form which might persist in the tissues for months and so give rise to relapses. The especial preference which the diplococcus shows for connective tissue, tendon, and muscle may account for the conditions met with in chronic or muscular rheumatism. The nature of the acid formed by the diplococcus is a matter still in dispute—lactic, formic, and acetic acids having each been named. The specific infective nature of acute rheumatism is not admitted by Stengel.[4] He inclines to the view that acute rheumatism should be regarded as an infectious arthritis which may be excited by several varieties of micro-organisms. He point out that the joint lesions are only those which might be expected to follow the intravenous injection of strepto-cocci or staphylococci; that different observers have isolated widely different species of cocci and bacilli, and that the lesions of acute articular inflammation are much alike, whatever organism be the exciting cause. Thus it is often difficult to distinguish between acute rheumatism and mild non-suppurative cases of joint affection following scarlet fever, enteric fever, small-pox, dysentery, pneumonia and pyæmia. Polyarticular chronic rheumatism of joints and tendons he thinks may be due to infective material thrown off from a previously infected endocardium. This view would seem to be negatived by observations lately made by Poynton and Shaw.[5] They obtained an attenuated culture of staphylococcus aureus by growing it in acid broth until it would produce no effects upon animals. This culture was then injected intravenously into rabbits and control rabbits were inoculated with the diplococcus rheumaticus. The staphylococcus injections produced either no results or a pyæmia with multiple abscesses; the diplococcus produced either rheumatic lesions or no effect at all. It was possible to produce a mixed effect by injecting both organisms at the same time, and in this case both rheumatic and pyæmic lesions resulted, and both organisms were recovered from the tissues after death, the staphylococcus being most readily found in the blood and the diplococcus in the joints. These results would seem to indicate that acute rheumatism is not an attenuated pyæmia. In the 25 fatal cases of rheumatic fever investigated the staphylococcus aureus could not be found.

**The Passage of Tubercle Bacilli through the Intestinal Wall.**—Ravenel[6] states that animals infected by feeding with tuberculous material may show extensive involvement of the lung when the intestines are either free of lesions or only slightly infected. Dobroklonski showed that the tubercle bacillus could pass through the healthy gut wall, and this has recently been confirmed by Nicholas[7] and Descos. Desoubry and Porcher have demonstrated that bacteria pass with the chyle through the gut wall, and Nocard says that during certain periods of digestion bacteria may be detected in the blood of horses. Revenel's experiments were made on dogs. After purging and a period of fasting an emulsion of tubercle bacilli, in butter and warm water, was introduced into the stomach. Four hours later the dogs were killed, and the chyle and mesenteric glands were examined for tubercle bacilli by means of inoculation experiments on guinea-pigs. In 8 out of 10 cases the results were positive, and the two negative results were probably due to the use of a very old culture of human tubercle. Of 24 guinea-pigs inoculated 21 showed microscopic and macroscopic evidences of tuberculosis. In no case could any trace of an intestinal lesion be found in the dogs. From these experiments he claims that, during the digestion of fat, tubercle bacilli may pass through the intact intestine with the chyle, enter the thoracic duct and venous system, and excite a primary pulmonary tuberculosis.

1 Jour. Path. and Bact, December, 1903. 2 Jour. of Hygiene, April. 1903. 3 Practitioner, January, 1901. 4 Amer. Jour. Med. Sci., March, 1903. 5 Brit. Med. Jour., Jan. 9, 1904. 6 Jour. Med. Research, December, 1903. 7 Jour. de Physiolog. et Path. Gen. IV., 1902.

